# Transosseous all-suture anchor fixation for bony Bankart lesions: a biomechanical study

**DOI:** 10.1186/s12891-025-09439-5

**Published:** 2025-12-23

**Authors:** Wen-Hao Chang, Fa-Chuan Kuan, Yueh Chen, Chih-Kai Hong, Wei-Ren Su, Kai-Lan Hsu

**Affiliations:** 1 Department of Medicine, College of Medicine, National Cheng Kung University, Tainan, Taiwan; 2 Department of Orthopaedic Surgery, College of Medicine, National Cheng Kung University Hospital, National Cheng Kung University, Tainan, Taiwan; 3 Skeleton Materials and Bio-compatibility Core Lab, Research Center of Clinical Medicine, College of Medicine, National Cheng Kung University Hospital, National Cheng Kung University, Tainan, Taiwan; 4 Department of Orthopedics, Kaohsiung Veterans General Hospital Tainan Branch, Tainan, Taiwan; 5 Department of Orthopaedic Surgery, College of Medicine, National Taiwan University Hospital, National Taiwan University, Taipei, Taiwan

**Keywords:** All-suture anchor, Biomechanical study, Metallic anchor, Bony Bankart lesion

## Abstract

**Background:**

The use of all-suture anchors (ASA) for treating bony Bankart lesions remains challenging because of the limited availability of cortical bone for secure fixation. In this study, the mechanical properties of transosseous ASA fixation, wherein the anchor is placed through the far cortex, were compared with those of metallic suture anchors and standard ASA fixation.

**Methods:**

For this controlled experimental study, 24 suture anchors were inserted into 12 synthetic scapulae with simulated bony Bankart lesions, with two anchors placed in each scapula. The scapulae were randomly assigned to Group A (two 2.7-mm metallic anchors), Group B (two 1.3-mm ASAs secured through standard fixation), or Group C (two 1.3-mm ASAs secured transosseously). For biomechanical analysis, a 5 N preload was applied for 2 min, followed by cyclic loading from 5 to 25 N at 1 Hz for 100 cycles. In addition, a load-to-failure test was conducted at a rate of 60 mm/min. Displacement, stiffness, ultimate failure load, and failure mode were recorded.

**Results:**

There were no significant differences and low correlations in biomechanical properties between the two anchors placed in each scapula. Nor significant intergroup differences were noted in cyclic displacement or linear stiffness. Group C exhibited a significantly higher (*P* < 0.001) ultimate failure load (176.8 ± 32.7 N) than did with Group A (104.4 ± 17.5 N) and Group B (83.0 ± 10.8 N). All failures resulted from anchor pullout, except for two cases in Group C, where deformed suture balls were observed without pullout.

**Conclusion:**

Compared with metallic suture anchors and standard ASA fixation, transosseous ASA fixation significantly improved the maximum failure load in a bony Bankart lesion model, without increasing cyclic displacement.

**Level of evidence:**

Basic Science Study; Biomechanics.

**Supplementary Information:**

The online version contains supplementary material available at 10.1186/s12891-025-09439-5.

## Introduction

A bony Bankart lesion refers to the detachment of the anteroinferior glenoid labrum, often accompanied by bone loss along the anterior glenoid rim. This condition often follows recurrent traumatic dislocations of the anterior shoulder. Of late, arthroscopic surgery has become the preferred treatment modality for bony Bankart lesions. Currently, arthroscopic repair using suture anchors is regarded as the standard approach for small-to-medium-sized bony Bankart lesions [1–3].

In recent years, all-suture anchors (ASAs) have increasingly been used in Bankart lesion repair. Unlike conventional metallic or bioabsorbable anchors, ASAs are entirely suture-based and achieve fixation through suture knot expansion during retraction. The advantages of ASAs include a small size, low bone damage during implantation, and high flexibility in implantation angles. Lee et al. [4] demonstrated that ASAs yield clinical outcomes similar to those of conventional anchors in patients with Bankart lesions. Likewise, Yanke et al. [5] found that in cyclic loading tests, ASAs exhibited elongation, stiffness, and ultimate failure load similar to those of conventional anchors. Despite their benefits, ASAs have certain limitations, given that their fixation depends on suture expansion beneath the cortical bone. If cortical integrity is compromised, stability may be affected. Ruder et al. [6] reported that in rotator cuff repair, decortication of the greater tuberosity considerably reduced ASA tensile strength. Under conditions characterized by cortical deficiency, such as bony Bankart lesions, ASAs may be susceptible to loosening.

To address this challenge, we explored transosseous ASA fixation as an alternative strategy. In this technique, the ASA is passed through the far cortical bone to leverage its greater structural integrity for better stability. Aramberri-Gutierrez et al. [7] used this technique for rotator cuff repair; the surgeons anchored ASAs through the far cortical bone and secured them against the inferomedial edge of the humeral head. Compared with conventional ASA placement in the near cortex, this approach significantly increased failure load and the number of cycles to failure, enhancing fixation strength and durability [8]. However, few studies have evaluated the efficacy of transosseous ASA fixation in bony Bankart repair.

In this study, we compared biomechanical properties among conventional anchor fixation with metallic sutures, standard ASA fixation, and transosseous ASA fixation in patients with bony Bankart lesions. We hypothesized that transosseous ASA fixation would result in a higher ultimate failure load than would metallic anchor fixation and standard ASA fixation.

## Materials and methods

### Model preparation

**Fig. 1:**
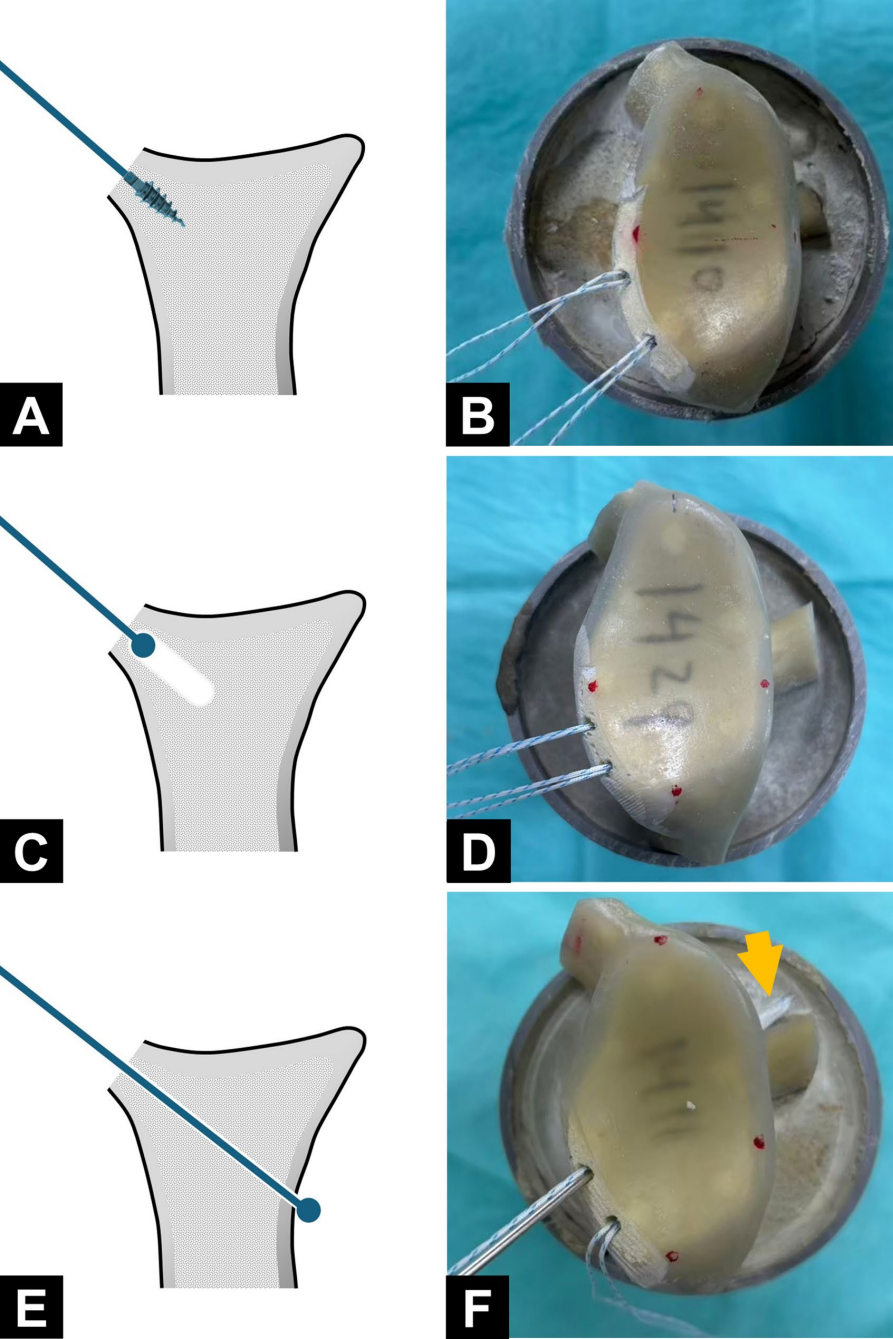
Illustrations and actual constructs of specimens from the study groups. **A** and **B** Group (A) **C** and **D**, Group (B) **E** and **F**, Group (C) In Group C, the all-suture anchor was passed through the far cortex of the glenoid (arrow)

**Fig. 2:**
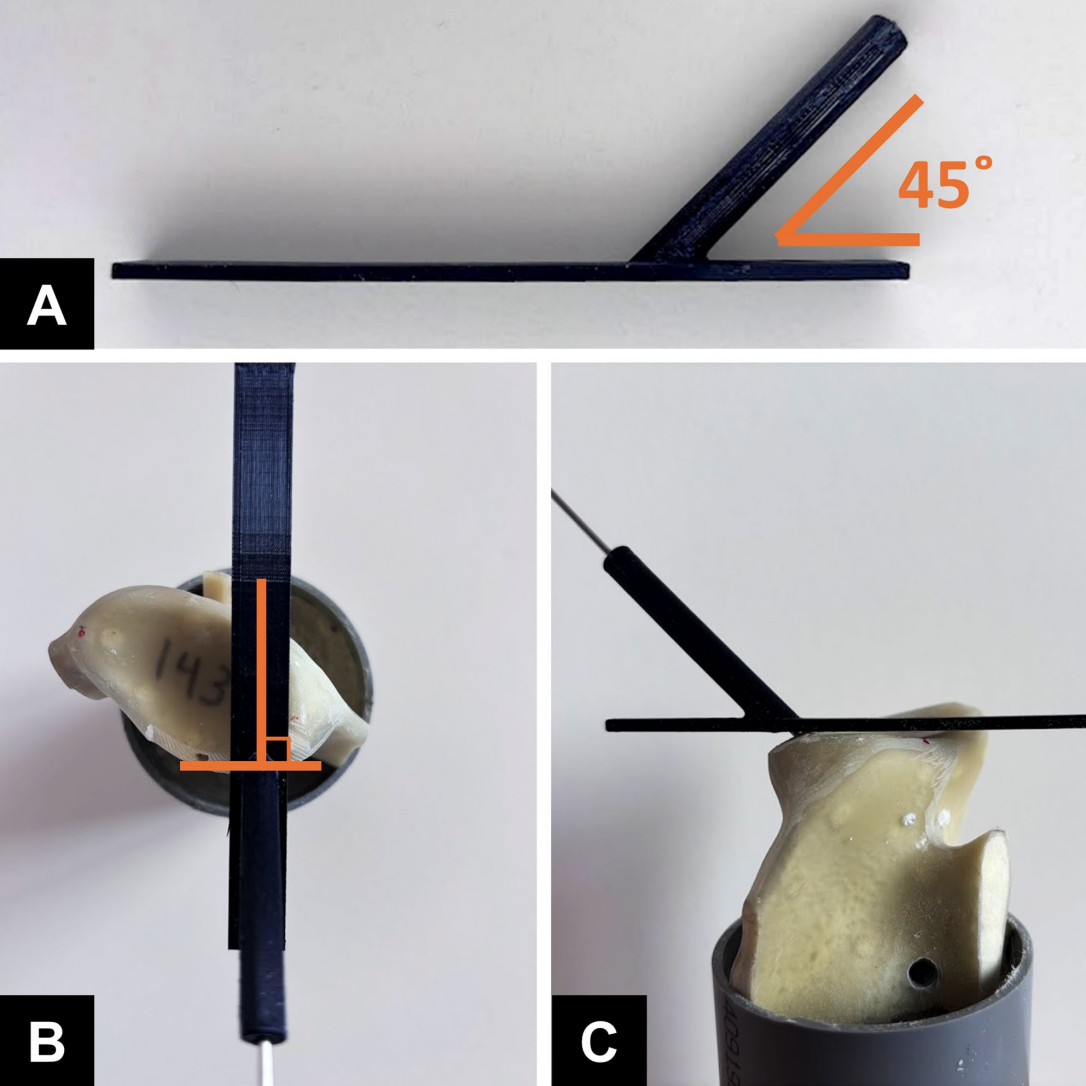
Use of the 3D-printed guiding jig in Group C. **A** The 3D-printed guiding jig. **B** The drilling trajectory oriented at 45° to the glenoid face in the axial plane. **C** The drilling trajectory aligned vertically with the edge of the bone defect in the sagittal plane

The study used an anchor pullout model in a simulated bony Bankart defect. Twenty-four suture anchors with eight metallic anchors (Mini Revo; 2.7 mm; ConMed Linvatec) and sixteen ASAs (Y-Knot; 1.3 mm; ConMed Linvatec) were used and each as an experimental unit. Twelve synthetic scapulae (Sawbones, Pacific Research Laboratories, Vashon, WA, USA) were used in this study. To facilitate mounting, the acromion, the coracoid process, and a portion of the scapular body were resected. Each scapula was then secured in a 6-cm polyvinyl chloride cylinder tube by using bone cement, with the glenoid surface aligned perpendicular to the tube axis and positioned approximately 5 cm above the cement surface. To simulate a bony Bankart lesion, cortical bone was removed from the anteroinferior glenoid rim, extending from the 6 o’clock to the 9 o’clock position (left side), exposing the underlying cancellous bone. Tewlve scapula were randomly assigned to three groups—Group A, Group B, and Group C, each undergoing anchor insertion performed using a distinct technique. In Group A, two 2.7-mm self-tapping metallic anchors were used (Fig. 1A, B). In Group B and Group C, two ASAs were used. In Group B, drill holes were prepared using the standard technique, and the anchors were secured beneath the subchondral bone (Fig. 1C, D). In Group C, drill holes were created using a 3D-printed guiding jig (Fig. 2A). The drilling trajectory was oriented at 45° to the glenoid face in the axial plane (Fig. 2B) and aligned vertically with the edge of the bone defect in the sagittal plane (Fig. 2C). Each drill hole was advanced through the far cortex, allowing the anchors to be securely fixed beneath it. (Fig. 1E, F). Across the groups, the anchors were placed at the 7 and 8 o’clock positions on the glenoid (left shoulder) at an insertion angle of 45° relative to the glenoid surface. All procedures for each scapula were performed by the same junior surgeon (WHC) with supervision from a senior surgeon (KLH).

### Biomechanical analysis

**Fig. 3:**
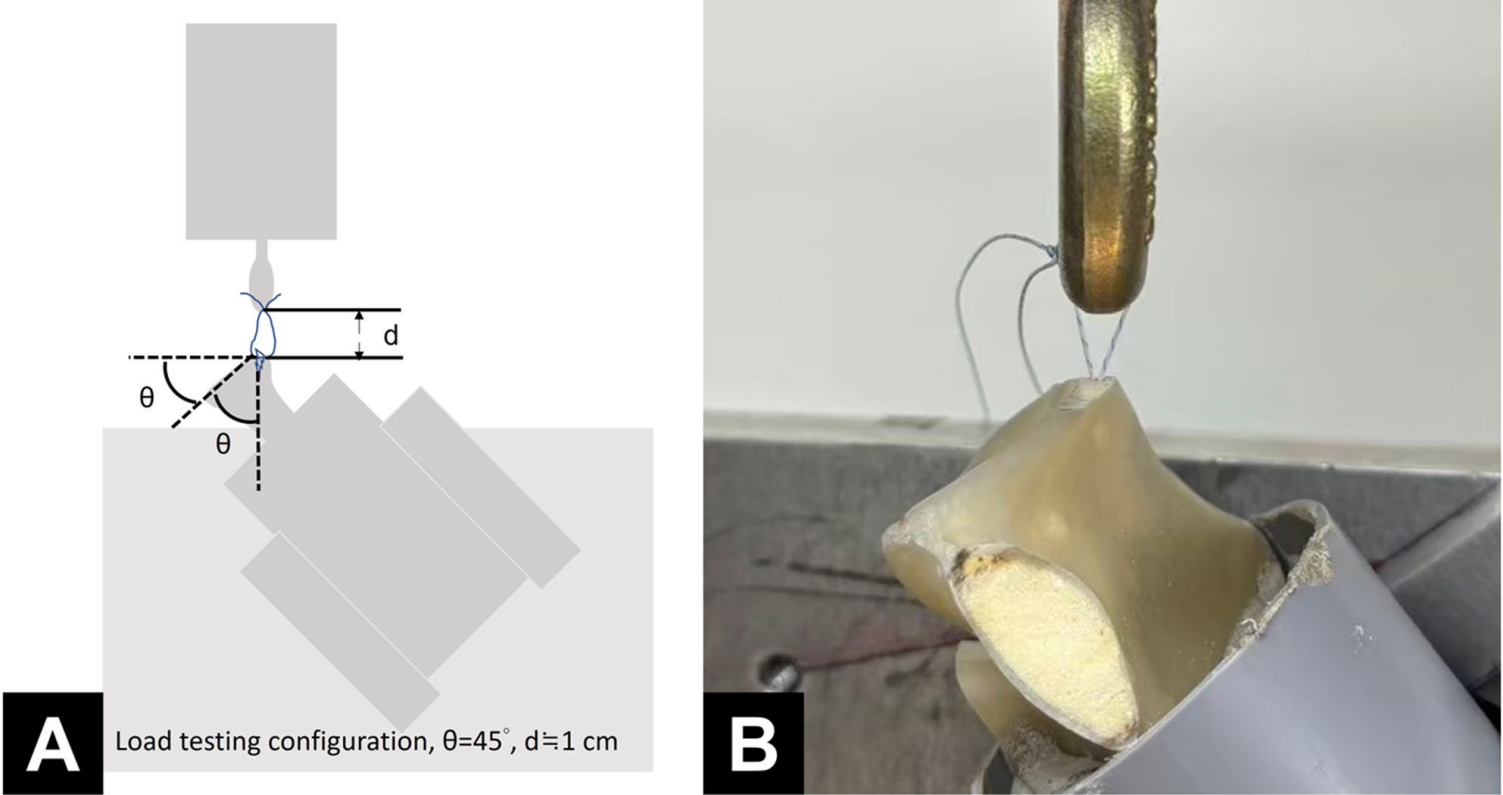
Illustration (**A**) and actual setup (**B**) for biomechanical analysis

Each scapula was positioned with the glenoid face at a 45° angle to the base of a material testing machine (AG-X; Shimadzu, Kyoto, Japan) and aligned so that the applied force was parallel to the anchor insertion axis. Sutures were tied with square knots and looped around the machine, maintaining a 1-cm gap (Fig. 3). Biomechanical analysis was performed in three phases: a 5 N preload for 2 min, cyclic loading between 5 and 25 N at 1 Hz for 100 cycles, and a load-to-failure test at a rate of 60 mm/min [5, 9, 10]. Failure was defined as a sudden drop in the force–displacement curve. Displacement during cyclic loading, linear stiffness, ultimate failure load, and failure mode were recorded. To ensure consistency, the biomechanical data were independently assessed by two observers, and any discrepancies were resolved by a third observer, who made the final determination.

### Statistical analysis

An a priori power analysis was performed using G*Power to determine the sample size required for comparing ultimate failure load among the three groups. On the basis of pilot data (*n* = 3 per group) and at an alpha level of 0.05 and a power of 0.80, the analysis revealed the need for approximately eight experimental unit per group. Given that each scapula could accommodate testing of two anchors, only four scapula per group were required to meet the testing criteria.

Statistical analyses were performed using SPSS (version 22; IBM, Armonk, NY, USA). Data are presented as mean ± standard deviation values. To assess potential clustering of measurements within each scapula, cyclic displacement, linear stiffness, and ultimate failure load were compared between the upper and lower anchors using paired tests, and Pearson correlation coefficients were calculated for each group. Intergroup differences in cyclic displacement, construct stiffness, and ultimate failure load were analyzed using the one-way analysis of variance (ANOVA) test, followed by the post hoc Tukey test when required. For the primary outcome (failure load), we additionally calculated standardized mean differences (Cohen’s d) using pooled standard deviations to describe the magnitude of improvement of construct C compared with constructs A and B. A *P* value of < 0.05 was considered statistically significant.

## Results

Each group comprised eight anchors, tested in four scapulae, with two anchors inserted into each scapula. In total, 24 anchors were tested in 12 scapulae across the three groups.

For comparison of the biomechanical properties between the upper and lower anchors within each scapula, cyclic displacement, linear stiffness, and ultimate failure load did not differ significantly between the two positions across all three groups. Pearson correlation coefficients between the two anchor positions within the same scapula were low and non-significant (r ranging from − 0.20 to 0.47) (Table 1).

**Table 1:** Comparison of Biomechanical properties between upper and lower anchors within the same scapula in each group

	Upper anchor (*n* = 4)	Lower anchor (*n* = 4)	*p*-value	Pearson correlation
Cyclic displacement, mm				
Group A	0.7 ± 0.3	1.3 ± 0.7	0.479	-0.097
Group B	0.7 ± 0.1	0.3 ± 0.2	0.272	-0.072
Group C	0.6 ± 0.2	0.9 ± 0.4	0.382	0.121
Linear stiffness, N/mm				
Group A	60.5 ± 13.2	51.2 ± 25.3	0.826	-0.198
Group B	44.1 ± 9.0	58.8 ± 15.3	0.203	0.467
Group C	57.1 ± 8.2	54.0 ± 14.2	0.399	0.378
Ultimate failure load, N				
Group A	109.4 ± 20.7	99.3 ± 7.5	0.456	0.107
Group B	84.4 ± 10.7	81.7 ± 9.3	0.755	0.338
Group C	188.0 ± 26.7	165.6 ± 30.1	0.371	0.232

Group A, metallic anchor fixation

Group B, standard all-suture anchor fixation

Group C, transosseous all-suture anchor fixation

**Fig. 4:**
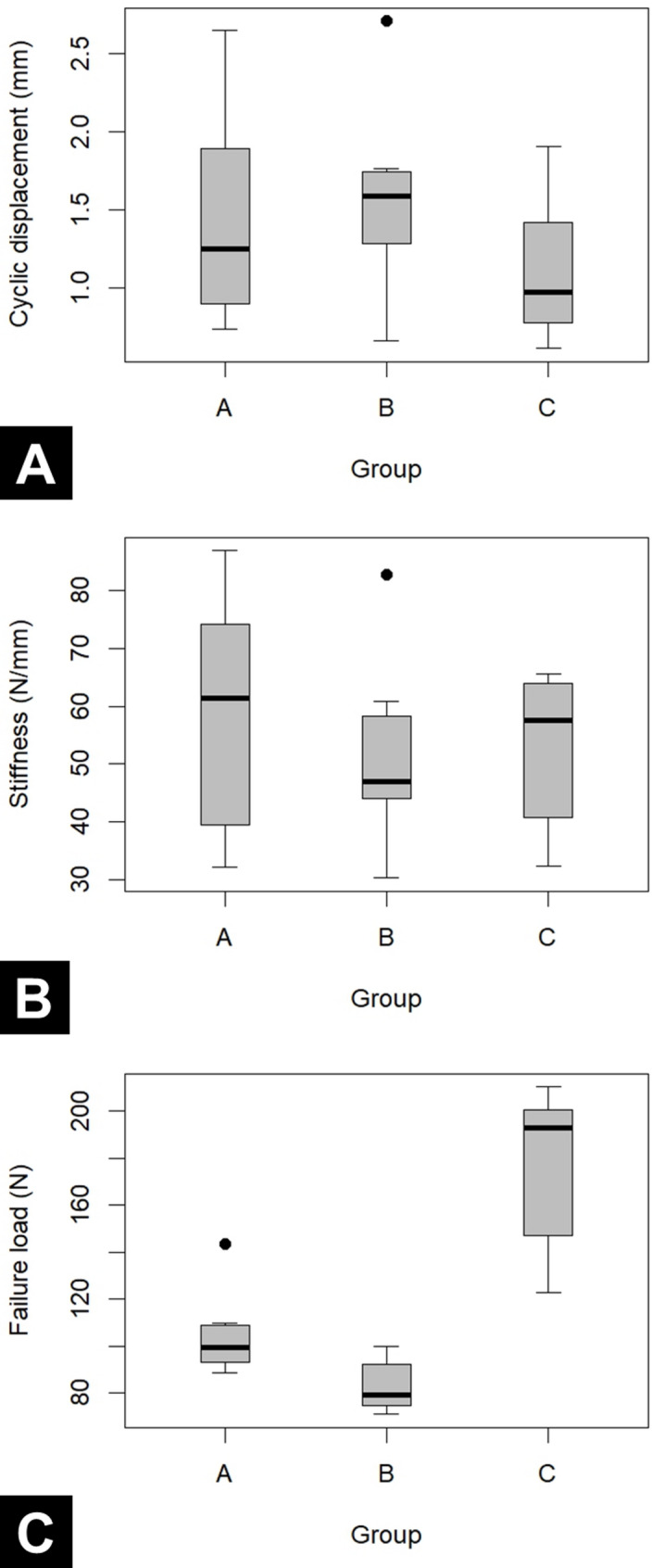
Box plot depicting displacement during cyclic loading **A**, linear stiffness **B**, and ultimate failure load (**C**) in each group. Group A, metallic anchors fixation; Group B, standard all-suture anchor fixation; and Group C, transosseous all-suture anchor fixation

For cyclic displacement, the differences between groups were very small, with all mean differences ≤ 0.21 mm and 95% CIs (Confidence interval) crossing zero, indicating only minimal effects. Similarly, for linear stiffness, the mean differences between groups were modest and not statistically significant, again suggesting only small effects (Table 2, Fig. 4A, B**)**. In contrast, group C showed a marked improvement in failure load compared with both group A and B. The mean failure load of group C (176.8 N) was 72.5 N higher than group A (104.4 N), with a 95% CI of 44.3 to 100.6 N (*P* < 0.001), corresponding to an approximate 69% increase in failure load (C vs. A).Compared with group B (83.0 N), group C showed a 93.8 N higher mean failure load, with a 95% CI of 65.7 to 121.9 N (*P* < 0.001), representing about a 113% increase (C vs. B). (Tables 2 and 3, Fig. 4C**)**.

**Table 2:** Displacement during Cyclic loading, linear stiffness, and ultimate failure load across the groups

Characteristics	Group A	Group B	Group C	*p* - value
Cyclic displacement, mm	0.9 ± 0.6	0.7 ± 0.2	0.8 ± 0.4	0.898
Linear stiffness, N/mm	58.7 ± 20.8	51.4 ± 15.5	52.8 ± 13.1	0.692
Ultimate failure load, N	104.4 ± 17.5	83.0 ± 10.8	176.8 ± 32.7	< 0.001

Group A, metallic anchor fixation

Group B, standard all-suture anchor fixation

Group C, transosseous all-suture anchor fixation

**Table 3:** Pairwise comparisons and effect sizes for ultimate failure load (Tukey HSD)

Comparison	Mean difference	95% CI	*p*-value
Group C vs. Group A	72.5	44.3 to 100.6	< 0.001
Group C vs. Group B	93.8	65.7 to 121.9	< 0.001
Group A vs. Group B	21.4	−6.7 to 49.5	0.159

*CI* Confidence interval

Group A, metallic anchor fixation

Group B, standard all-suture anchor fixation

Group C, transosseous all-suture anchor fixation

All anchors in Group A and Group B failed due to suture anchor pullout. However, variations were observed in the mode of failure in Group C: six out of eight anchors (75%) exhibited suture tip migration into the bone tunnel at the time of construct failure, whereas the remaining 2 anchors (25%) retained suture tips against the far cortex, although with visibly deformed suture balls. Load–displacement curves from Group C indicated successful bicortical fixation with ASAs.

## Discussion

The main finding of this study was that transosseous ASA fixation significantly increased the maximum failure load compared with both metallic suture anchors and standard ASA fixation in an anchor pullout model with a simulated bony Bankart defect, without increasing cyclic displacement.

In the treatment of bony Bankart lesions, cortical deficiency poses a significant challenge to the surgeon. Metallic suture anchors can provide adequate fixation strength in cancellous bone; however, anchor pull-out or prominence may lead to articular cartilage damage [11, 12]. Double-row repair using multiple anchors has been proposed to address this problem and can yield satisfactory clinical outcomes [10, 13], but the technique is technically demanding and increases cost due to the greater number of anchors required [14, 15]. Bony augmentation procedures represent another treatment option, yet their indication in cases with relatively small bony defects remains controversial [16, 17]. With the increasing prevalence of ASA use in Bankart lesions, the effectiveness of ASA in the management of bony Bankart lesions warrants further evaluation.

The fixation strength of ASAs depends heavily on cortical bone thickness. In a biomechanical study, a mean decortication of 1.7 mm at the greater tuberosity significantly reduced the ultimate failure load of ASA fixation [18]. In patients with bony Bankart lesions, anteroinferior cortical bone deficiency increases the risk of anchor pullout when ASAs are used. To address this challenge, some surgeons place the anchor on the articular surface [19, 20]. However, this approach may reduce the proportion of native glenoid surface area restored [21] and increase the risk of glenoid rim erosion compared with the risk in anchor placement at the glenoid edge [22]. Other surgeons have used double-row repair techniques to avoid anchoring into cancellous bone [15, 23], but this method is technically demanding and economically burdensome. Qian et al. [24] introduced an inverted ASA fixation technique, which involves passing the anchor through the bone tunnel and securing it on the fragment cortex rather than beneath the glenoid edge. Although this technique is promising, it presents some technical challenges. In the present study, ASAs were placed at the glenoid edge and anchored through the far cortex. This technique is similar to that used for single-row repair [25, 26], aligning with common surgical practice. The ultimate failure load of the transosseous ASA technique was similar to that of standard ASA placement in the intact glenoid without bone loss [27, 28]. Moreover, this load was higher for transosseous ASA fixation than for conventional ASA fixation and metallic anchor fixation.

However, the increased suture length between the anchor and the bony edge in the transosseous ASA technique remains a matter of concern. Elongation due to elastic stretching is proportional to suture length [29]. This leads to considerable cyclic displacement when the transosseous ASA technique is used. Cyclic loading tests are commonly conducted in biomechanical studies to simulate rehabilitation activities [30]. In our study, the cyclic testing protocol was set to range from 5 to 25 N for 100 cycles, representing at least 10% of the ultimate load and replicating physiological loading conditions in a mild postoperative rehabilitation setting [31]. Evidence suggests that displacement exceeding 1.5 mm leads to clinical failure [32]. In the present study, the transosseous ASA fixation technique resulted in a cyclic displacement similar to that noted with other fixation methods. The mean cyclic displacement with transosseous ASA fixation remained below 1.5 mm. Therefore, this technique is a safe and viable, offering acceptable cyclic displacement within clinically appropriate limits.

**Fig. 5:**
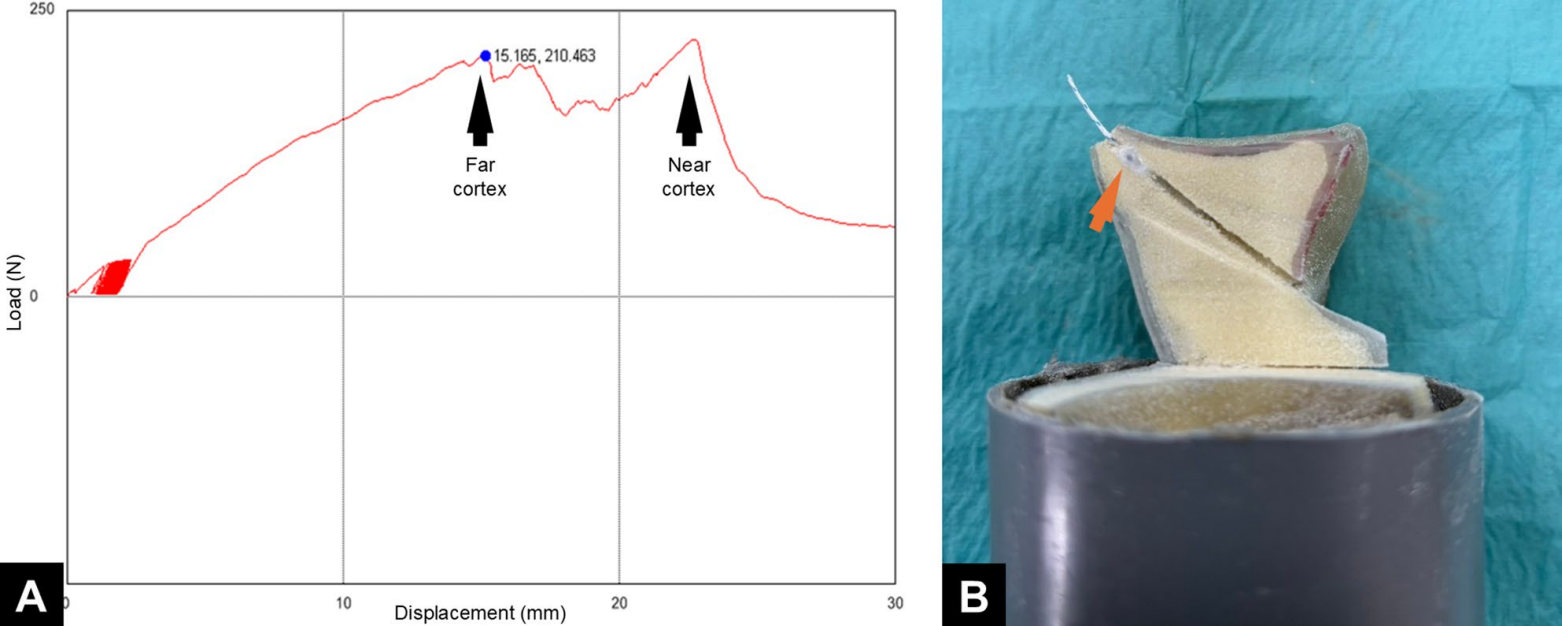
**A** Load–elongation curve for a specimen fixed with transosseous all-suture anchor fixation exhibited characteristics consistent with those noted for bicortical fixation. **B** Another specimen, when dissected, exhibited anchor engagement near the bony edge (arrow)

Although transosseous ASA fixation led to the highest ultimate failure load, it was also associated with a large standard deviation and coefficient of variation. This variability may be attributable to the challenges surgeons face when inserting the ASA through the far cortex. The current design of the soft drill and drill guide for ASA is intended primarily for creating a pilot hole of appropriate depth in the near cortex. However, when drilling through the far cortex is necessary, the soft drill and guiding jig design is often unsuitable for pilot hole creation and subsequent ASA insertion. This technical inconsistency may lead to anchor damage, contributing to high variability in ultimate failure load. The load–elongation curve for transosseous ASA fixation exhibited characteristics similar to those noted for bicortical fixation (Fig. 5A) [33], potentially engaging the near bony edge (Fig. 5B). However, given the considerable distance between the far cortex and the near bony edge, such engagement is clinically irrelevant.

**Fig. 6:**
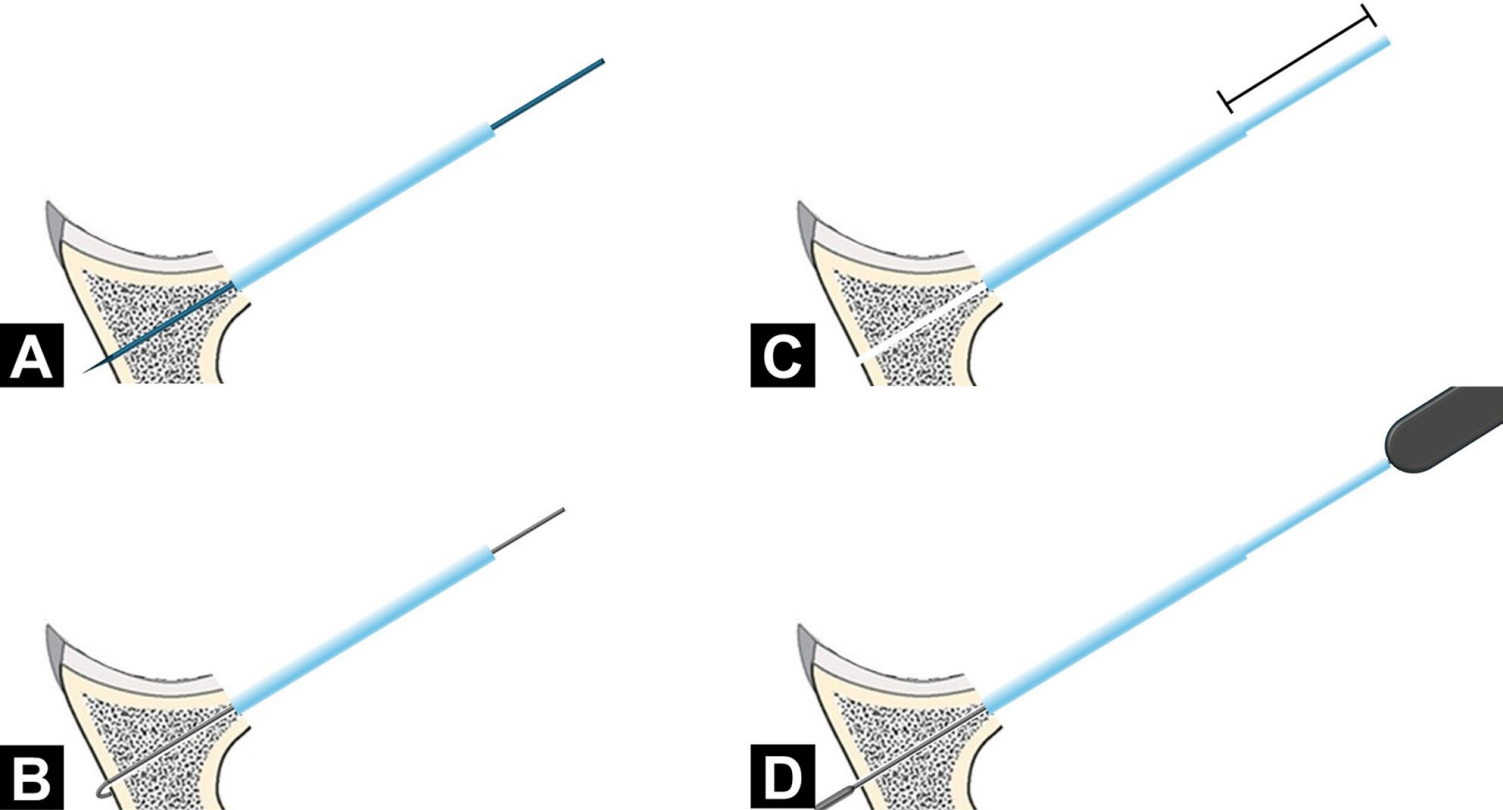
Concept of an adjustable guiding jig for transosseous all-suture anchor fixation. The proposed adjustable guiding jig allows controlled transosseous drilling and anchor placement. **A** A short guiding sheath is first used to create the bone tunnel. **B** After drilling, the tunnel depth is measured. **C** The adjustable guiding sheath is then extended to the specified length. **D** Finally, the all-suture anchor is inserted through the adjusted sheath so that it traverses the tunnel with a controlled depth, reducing the risk of over-penetration while maintaining reproducible bicortical fixation

Although transosseous ASA fixation showed promising biomechanical performance in this study, several technical concerns remain. First, the current ASA guiding jig provides a fixed tunnel length, whereas in the transosseous configuration the tunnel length is not constant, and the central ASA guide may bend or deflect if an inappropriate jig is used. An adjustable, transosseous-specific guiding jig may therefore be required to ensure reliable bicortical drilling (Fig. 6). Second, the risk of suprascapular nerve injury must be considered when the anchor guide is directed toward the posterior cortex [34]. According to the anatomical study by Ladermann et al. [35] the angle of the spinoglenoid notch relative to the glenoid surface in the axial plane is 24° ± 6°, which is substantially smaller than the 45° insertion angle used in the present model, suggesting a theoretical safety margin. Nevertheless, our experiments were performed in a synthetic model without soft tissue or neurovascular structures, so the in vivo relationship between a transosseous tunnel and the suprascapular neurovascular bundle remains uncertain. Further cadaveric or anatomical studies are needed to better define a safe zone for clinical application.

### Limitations

This study has several limitations. First, we evaluated only time-zero mechanical properties under a uniaxial testing configuration, which may not fully replicate the multidirectional and shear forces acting on the glenoid during shoulder motion, nor the dynamic biological environment present in vivo. Therefore, further clinical studies are required to determine how our biomechanical findings translate to actual bony lesion healing. Second, we used Sawbones biomechanical models to simulate human bone; However, the interface between cortical and cancellous bone in these models may differ from that of native human bone, and the heterogeneous distribution of bone density in the scapula [36] may introduce bias. In addition, the processes of drilling, anchor insertion, and the resulting biomechanical behavior are likely to differ between cadaveric specimens and synthetic sawbones. Third, operator-related bias may exist. The manual drilling of the far cortex introduces potential variability in trajectory and depth. Even with the use of a custom-made guide, small deviations in the drilling angle may influence cortical engagement and tunnel length, thereby contributing to variability in failure load. Finally, because this study focused solely on the biomechanical properties of the anchors, soft-tissue tensioning—such as that provided by the capsulolabral complex—as well as fragment compression, contact area, and labral tissue behavior were not reproduced in our model; therefore, the clinical performance of the entire repair construct may differ from our biomechanical findings.

## Conclusion

In this biomechanical study, the transosseous ASA fixation technique significantly increased the maximum failure load in a simulated bony Bankart lesion model, without increasing cyclic displacement. Our findings position this technique as a promising surgical option for bony Bankart lesions.

## Supplementary Information

Supplementary Material 1.

## Data Availability

All data used during the study are available from the corresponding author.

## Abbreviations

ASA: All-suture anchors
