# Supplementary material for: Transosseous all-suture anchor fixation for bony Bankart lesions: a biomechanical study
Source: BMC Musculoskelet Disord. 2025 Dec 23;27:61. doi: 10.1186/s12891-025-09439-5 (PMC12837074; doi:10.1186/s12891-025-09439-5)
Supplement: Supplementary file 1 — Supplementary Material 1. [file 12891_2025_9439_MOESM1_ESM.docx]

**Title page**

Original Article

**Transosseous All-Suture Anchor Fixation for Bony Bankart Lesions: A Biomechanical Study**

**Running title: All-Suture Anchor for Bony Bankart**

Wen-Hao Chang, MD ^1^, Fa-Chuan Kuan, MD, PhD ^2,3^, Yueh Chen, MD, PhD ^4^,

Chih-Kai Hong, MD ^5^, Wei-Ren Su, MD^2,3^, Kai-Lan Hsu, MD, PhD ^2,3^

1. Department of Medicine, College of Medicine, National Cheng Kung University, Tainan, Taiwan.
2. Department of Orthopaedic Surgery, National Cheng Kung University Hospital, College of Medicine, National Cheng Kung University, Tainan, Taiwan
3. Skeleton Materials and Bio-compatibility Core Lab, Research Center of Clinical Medicine, National Cheng Kung University Hospital, College of Medicine, National Cheng Kung University, Taiwan
4. Department of Orthopedics, Kaohsiung Veterans General Hospital Tainan Branch, Tainan, Taiwan
5. Department of Orthopaedic Surgery, National Taiwan University Hospital, College of Medicine, National Taiwan University, Taipei, Taiwan

**Wen-Hao Chang**

Department of Medicine, College of Medicine, National Cheng Kung University, Tainan, Taiwan.

Address: 138 Sheng-Li Rd., Tainan 701, Taiwan

Email : freddy70229@gmail.com

**Kai-Lan Hsu, Fa-Chuan Kuan, Wei-Ren Su**

Department of Orthopedics, National Cheng Kung University Hospital. Tainan, Taiwan

Address: 138 Sheng-Li Rd., Tainan 701, Taiwan

Email :

Kai-Lan Hsu: [dulendulen@gmail.com](mailto:dulendulen@gmail.com)

Fa-Chuan Kuan: justoversea@hotmail.com

Wei-Ren Su: suwr@ms28.hinet.net

**Yueh Chen**

Department of Orthopedics, Kaohsiung Veterans General Hospital Tainan Branch, Tainan, Taiwan

Address: No.427, Fuxing Rd., Yongkang Dist., Tainan 710, Taiwan

Email: b1729kimo@gmail.com

**Chih-Kai Hong**

Department of Orthopaedic Surgery, National Taiwan University Hospital, College of Medicine, National Taiwan University, Taipei, Taiwan

Chih-Kai Hong: [yayahong@gmail.com](mailto:yayahong@gmail.com)

**Correspondence**

Please address all correspondence to:

**Kai-Lan Hsu, MD**

Department of Orthopedics, National Cheng Kung University Hospital. Tainan, Taiwan

Address: 138 Sheng-Li Rd., Tainan, Taiwan, R.O.C

Email : [dulendulen@gmail.com](mailto:dulendulen@gmail.com)
